# Zeolite-silver-zinc nanoparticles: Biocompatibility and their effect on the compressive strength of mineral trioxide aggregate

**DOI:** 10.4317/jced.53392

**Published:** 2017-03-01

**Authors:** Mohammad Samiei, Negin Ghasemi, Naser Asl-Aminabadi, Baharak Divband, Yasamin Golparvar-Dashti, Sajjad Shirazi

**Affiliations:** 1 Associate Professor, Department of Endodontics, Faculty of Dentistry, Tabriz University of Medical Sciences, Tabriz, Iran; 2 Assistant Professor, Department of Endodontics, Faculty of Dentistry, Tabriz University of Medical Sciences, Tabriz, Iran; 3 Professor, Department of Pediatric Dentistry, Faculty of Dentistry, Tabriz University of Medical Sciences, Tabriz, Iran; 4 Associate Professor, Drug Applied Research Center, Tabriz University of Medical Sciences, Tabriz, Iran; 5 Under Graduate Student, Student Research Committee, Faculty of Dentistry, Tabriz University of Medical Sciences, Tabriz, Iran; 6 Research Fellow and Lecturer, Dental and Periodontal Research Center, Faculty of Dentistry, Tabriz University of Medical Sciences, Tabriz, Iran; 7 Nanotechnology Research Center, Tabriz University of Medical Sciences, Tabriz, Iran

## Abstract

**Background:**

This study was carried out to evaluate the biocompatibility of zeolite-silver-zinc (Ze-Ag-Zn) nanoparticles and their effect on the compressive strength of Mineral Trioxide Aggregate (MTA).

**Material and Methods:**

Biocompatibility was evaluated by an MTT assay on the pulmonary adenocarcinoma cells with 0.05, 0.1, 0.25, 0.5, 1 and 5 mg/mL concentrations of Ze-Ag-Zn. For compressive strength test, four groups containing 15 stainless-steel cylinders with an internal diameter of 4 and a height of 6 mm were prepared and MTA (groups 1 and 2) or MTA + 2% Ze-Ag-Zn (groups 3 and 4) were placed in the cylinders. The compressive strength was evaluated using a universal testing machine 4 days after mixing in groups 1 and 3, and 21 days after mixing in groups 2 and 4.

**Results:**

There was no significant difference between cytotoxicity of different concentrations. The highest (52.22±18.92 MPa) and lowest (19.57±5.76 MPa) compressive strength were observed in MTA group after 21 days and in MTA + 2% Ze-Ag-Zn group after four days, respectively. The effect of time and 2% Ze-Ag-Zn on the compressive strength were significant (*P*<0.05). Mixing MTA with Ze-Ag-Zn significantly reduced and passage of time from day four to 21 significantly increased the compressive strength.

**Conclusions:**

Mixing MTA with 2% Ze-Ag-Zn had an adverse effect on the compressive strength of MTA, but this combination had no cytotoxic effects.

** Key words:**Compressive strength, Cytotoxicity, Mineral Trioxide Aggregate, Nanoparticle, Zeolite-Silver-Zinc.

## Introduction

Mineral Trioxide Aggregate (MTA) is a calcium silicate-based hydrophilic cement which is widely used in endodontics for perforation repair and root canal treatment due to several favorable characteristics like biocompatibility and the capacity to induce osteogenesis and cementogenesis (1-3). However, its long setting time and handling difficulties are among its disadvantages (4,5). Various techniques have been proposed to improve the properties of MTA including the incorporation of different additives such as calcium chloride, Na2HPO4 and nanoparticles (6-9).

Silver-zeolite nanoparticle has been used to improve the properties of materials in dentistry. It is a crystalline structure of alumino-silicate with a porous molecular structure in which various ions such as zinc and silver is embedded in the pores (10,11). A review of the literature in relation to the use of zeolite in combination with various elements such as silver and zinc in the dental field indicates its positive antimicrobials effect (12,13). The cytotoxicity of endodontic sealer with 2 wt% of silver-zeolite is similar to that of glass-ionomer and is less than that of AH26 (12,13). Incorporation of silver-zeolite to MTA has resulted in an increase in its solubility and release of calcium and also in a decrease in setting time. In this context, mixing with a 2 wt% has led to better results than mixing with 0.2 wt% (10). In addition, a combination of MTA with silver-zeolite has resulted in an increase in its antibacterial activity against microorganisms such as *E. faecalis*, *S. aureus* and *C. albicans* (11).

The compressive strength of MTA is important when it is used to repair furcal perforations, pulp capping or apexogenesis where it must resist against being crushed as a result of occlusal forces and the placement of restorative materials (5). Compressive strength of hydraulic cement is as an indicator of its hydration reaction and in fact is indirectly an indication of the setting process of the material. This physical property is affected by the type of MTA, the liquid with which it is mixed, the condensing pressure and techniques used to mix the powder and liquid (14,15).

To the best of our knowledge, there are no published studies available on the effect of silver-zeolite-zinc (Ze-Ag-Zn) on the compressive strength of MTA. This study was designed to evaluate the effect of adding 2 wt% Ze-Ag-Zn on the compressive strength of MTA at two time intervals of 4 and 21 days after mixing. In addition, the cytotoxicity of Ze-Ag-Zn on the pulmonary adeno-carcinoma cells was evaluated.

## Material and Methods

The protocol for the study was independently reviewed and approved by the institutional review board.

-Preparation of Ze-Ag-Zn particles

HZSM-5 zeolite was synthesized in the laboratory. Zinc nitrate, AgNO3, was obtained from Merck (Merck, Darmstadt, Germany). The powder form ZSM-5 was modified by liquid phase ion exchange (LPIE), using Ag+ and Zn2+ cations. First, Ag (I)-ZSM-5 was prepared by a 24-h ion exchange with a solution of 10g of ZSM-5 and 300mL of silver nitrate (1M) at ambient temperature. Then, 10g of Ag (I)-ZSM-5 were added to 300mL of zinc nitrate solution (5M) for preparation of Ag (I) and Zn (II)-ZSM-5. After each exchange process, the modified zeolite suspension was filtered and washed with copious amounts of deionized water (16).

-Characterization of Ze-Ag-Zn particles

X-ray diffraction patterns (XRD) were collected using a Siemens D500 diffractometer with Cu kα radiation (λ=1.5418 Aº and θ=4-80º) at room temperature. Scanning electron microscope (Philips XL30) equipped with energy dispersive X-ray (EDX) facility was used to capture SEM images and to perform elemental analysis. The SEM sample was gold coated prior to examination and SEM was operated at 5kV while EDX analysis was performed at 15 kV. TEM studies, combined with EDX were carried out on a Zeiss LEO 912 Omega instrument, operating at 120 kV. TEM specimens were made by evaporating one drop of solution of sample in ethanol onto carboncoated copper grids. Grids were blotted dry on filter paper and investigated without further treatment. The determination of aluminum, silicon, zinc and silver contents in zeolite samples was carried out using standard inductively coupled plasma (ICP) method. The samples for ICP analysis were prepared by fusing the modified zeolite with sodium hydroxide.

-MTT assay for cell viability

A549 alveolar adenocarcinoma cells (8×103 cells/well) were incubated in 96-well plates, each containing 200µL of supplemented cell culture media for 24 hours at 37°C and 5% CO2. The cells were divided into 7 groups (n=12): control, Ag (I) and Zn (II)-ZSM-5 (different concentrations of 0.05, 0.1, 0.25, 0.5, 1 and 5mg/mL) were treated. After an incubation period of 24h, the used media was removed and the plate wells were washed with phosphate-buffered solution. Then, 50μL of 2mg/mL of MTT (3-(4, 5-dimetylthiazol-2-yl)-2, 5-diphenyl-trazolium bromide) and 150μL of culture medium were added to each well. The cells were incubated at 37°C and 5% CO2 for 4 h and then the media was discarded and dimethyl sulfoxide and Sorenson buffer were added to each well as a solubilizer buffer. Finally, absorbance was read using an ELISA plate reader (BioTeck, Bad Friedrichshall, Germany) at a wavelength of 570nm (17,18).

-Preparation of samples for compressive strength test

Based on the experimental material and the assessment time of compressive strength, 4 groups were considered in this study. For each group of the study, 15 steel cylinders with an internal diameter of 4mm and a height of 6mm were prepared. In groups 1 and 2, they were filled with MTA (Angelus, Londrina, Brazil) and in groups 3 and 4, they were filled with MTA + 2% Ag-Zn-Ze.

-Measurement of compressive strength 

The compressive strengths were determined in accordance with ISO 9917-1 method. Each material was mixed and placed in a split stainless steel mold (4.0mm in inner diameter and 6.0mm in height). The mixed cement was then compacted into each mold using a spatula and further compacted using a dental plugger to ensure a dense and uniform sample with minimal porosity. Once filled, the excess material was scraped off with the edge of a glass microscopic slide to leave a flat and uniform surface. No later than 120s after mixing, the complete assembly was transferred into an incubator maintained at 37°C for 6h, after which they were removed from the molds and checked visually for any air voids or chipped edges.

Fifteen sound samples were prepared for each group and placed in closed containers containing a piece of gauze impregnated with phosphate-buffered saline solution. Compressive strength assessment was performed using a universal testing machine (Hounsfield Test Equipment, Redhill, Surrey, UK) 4 days (groups 1 and 3) and 21 days (groups 2 and 4) after mixing. Force was applied at a crosshead speed of 1mm/min parallel to the long axis of the molds until the materials were crushed. The force was recorded in Megapascals (MPa) 

-Statistical analysis

The Kolmogorov-Smirnov test and Q-Q plot were used to check the normal distribution of the data. Levene’s test was used to assess the equality of variances (19,20). After calculating the mean ± standard deviation of compressive strength, one-way ANOVA was used to assess the significance of the effect of time and the type of material (with and without Ag-Zn-Ze) on the compressive strength (21). Post hoc Tukey test was used for pairwise comparisons of the groups (22). Data on cell viability was analyzed with T-test (17). *P*<0.05 was considered statistically significant.

## Results

-Characteristics of Ze-Ag-Zn particles

**Figure 1:**
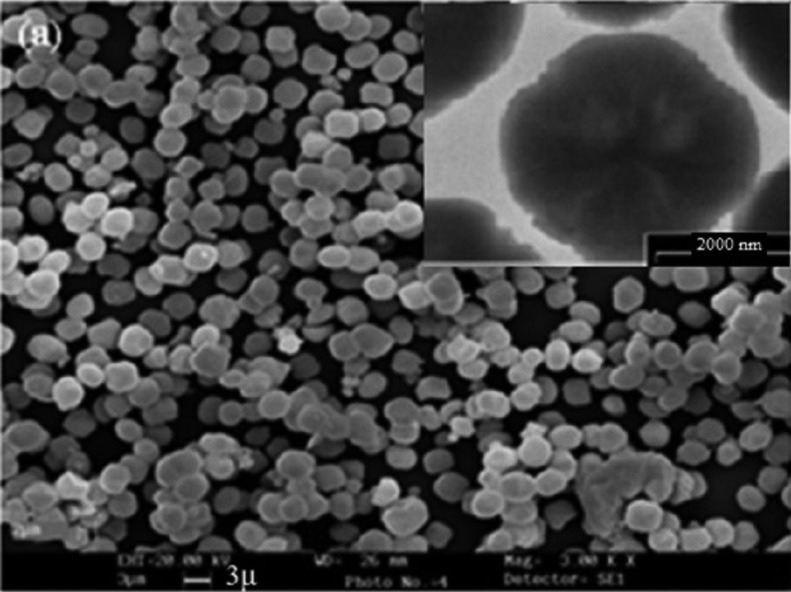
The size and morphology of Ze-Ag-Zn particles. Insert: TEM image.

The size and morphology of Ag (I) & Zn(II)-ZSM-5 is illustrated in figure 1. This composite has spherical to cubical shape crystals. No amorphous phase is observable, indicating high purity of the sample. The average of the particle diameter of the composite (Fig. 1, TEM image, insert) is about 2-3 μm.

-Cytotoxicity of Ag (I) and Zn (II)-ZSM-5

**Figure 2:**
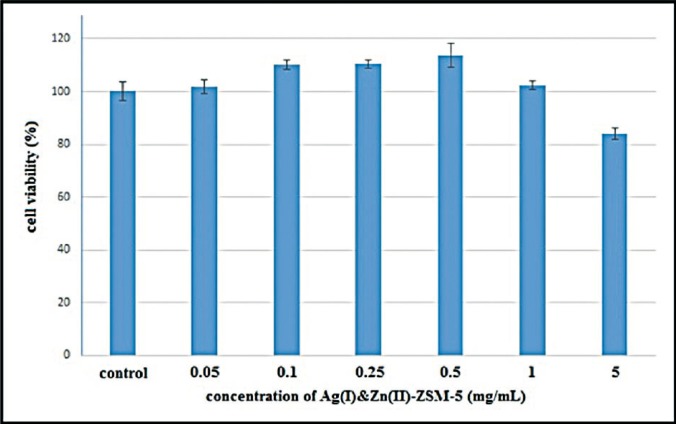
Cell viability after exposure to Ze-Ag-Zn particles.

The relative viability of cells treated with 0.05, 0.1, 0.25, 0.5, 1 and 5mg/mL concentrations of Ag (I) & Zn (II)-ZSM-5 after 24 hours of incubation is presented in figure 2. T-test showed no significant difference in relation to cytotoxicity in the study groups (*P*<0.05).

-Compressive strength

The highest and lowest compressive strength was obtained in MTA group after 21 days (52.22±18.92MPa) and MTA + Ze-Ag-Zn group after 4 days (19.57±5.76MPa), respectively. Compressive strength was 35.85±12.34MPa for MTA group after 4 days and 32.78±9.43MPa for MTA + Ze-Ag-Zn group after 21 days.

ANOVA showed a significant effect of the presence or absence of Ze-Ag-Zn, and time on the compressive strength (*P*=0.03 and *P*=0.01, respectively). The presence of Ze-Ag-Zn significantly reduced the compressive strength (*P*<0.05). Regardless of the presence or absence of Ze-Ag-Zn, the passage of time from 4 to 21 days significantly increased the compressive strength (*P*=0.03 for MTA groups and *P*=0.01 for MTA + Ze-Ag-Zn groups). Pairwise comparisons of the studied groups showed significant differences between all groups (*P*<0.05).

## Discussion

This study aimed to assess the effect of Ze-Ag-Zn on the compressive strength of MTA. The Ze-Ag-Zn significantly reduced the compressive strength of MTA. Over time, the compressive strength of all groups in the study increased. The other finding of our study was the biocompatibility of Ze-Ag-Zn particles.

The cytotoxicity of materials should be evaluated before their clinical application. In the present study, MTT assay was used to evaluate the cytotoxicity of Ze-Ag-Zn. MTT is a colorimetric assay based on the ability of mitochondrial dehydrogenase in living cells to convert the yellow water-soluble tetrazolium salt into dark blue formazan crystals. The amount of formazan produced is directly proportional to the viable cell counts (23-25). Its advantage is that the formazan crystals are soluble in tissue culture media and therefore the solving procedure is omitted. In this study, viability of cells was determined based on the MTT assay because of its simplicity, precision and accessibility (4).

The initial compressive strength of MTA is 40 MPa and it reaches to 67 MPa after 21 days according to previous studies (14). Compressive strength indicates the quality of hydration process and increased amount of compressive strength over time in both groups in our study is consistent with the results of previous studies (5,15,26). This indicates that MTA was hydrated from day 4 to 21 and obtained its strength to some extent. In the method used in this study, both groups were kept in closed containers with gauze soaked in phosphate-buffered saline until clinical conditions were reconstructed and MTA was exposed to moisture.

In the present study, we chose two time periods to assess the compressive strength. The shorter time interval of 4 days was selected because the primary strength is important in clinical applications when the material is exposed to occlusal forces in the clinical situation. Also in this period, the material will reach optimum setting. Time interval of 21 days was chosen in order to investigate the effect of Ze-Ag-Zn, at a longer time interval in addition to the short time and compare the results with the control group (wit-hout Ze-Ag-Zn). Long-term strength is important for the material’s resistance against crushing caused by forces resulting from placing the restorative materials and occlusal forces (5).

In this study, attempts were made to control factors affecting the compressive strength of MTA. The powder-to-liquid ratio was the same in all the samples. Moreover, Ze-Ag-Zn was used at 2 wt% because in previous studies this percentage positively affected some properties of MTA (10,11). Mixing the powder with liquid was carried out manually by one person for all the samples for 15 seconds. Materials were later placed in the cylinder by one operator. In previous studies, the mechanical mixing and placement with ultrasonic agitation increased the compressive strength (27). However, in this study we used only the manual method to remove the interaction between adding Ze-Ag-Zn, and mixing and placement method because it was the first time that the incorporation of Ze-Ag-Zn and its effect on the compressive strength were investigated. Temperature and humidity were kept the same for all the samples.

## Conclusions

Under the limitations of this study, the use of MTA + Ze-Ag-Zn is not recommended in cases where the compressive strength is important, such as the repair of furcal perforation, pulp capping and apexogenesis. However, MTA with Ze-Ag-Zn can be used as an apical plug and retro-filling material in surgeries where compressive strength is not important.

## References

[1] Lotfi M, Ghasemi N, Rahimi S, Bahari M, Vosoughhosseini S, Saghiri MA (2014). Effect of smear layer on the push-out bond strength of two endodontic biomaterials to radicular dentin. Iran Endod J.

[2] Aminabadi NA, Huang B, Samiei M, Agheli S, Jamali Z, Shirazi S (2016). A Randomized Trial Using 3Mixtatin Compared to MTA in Primary Molars with Inflammatory Root Resorption: A Novel Endodontic Biomaterial. J Clin Pediatr Dent.

[3] Asl Aminabadi N, Satrab S, Najafpour E, Samiei M, Jamali Z, Shirazi S (2016). A randomized trial of direct pulp capping in primary molars using MTA compared to 3Mixtatin: a novel pulp capping biomaterial. Int J Paediatr Dent.

[4] Ghasemi N, Rahimi S, Lotfi M, Solaimanirad J, Shahi S, Shafaie H (2014). Effect of Mineral Trioxide Aggregate, Calcium-Enriched Mixture Cement and Mineral Trioxide Aggregate with Disodium Hydrogen Phosphate on BMP-2 Production. Iran Endod J.

[5] Shahi S, Ghasemi N, Rahimi S, Yavari HR, Samiei M, Janani M (2015). The effect of different mixing methods on the flow rate and compressive strength of mineral trioxide aggregate and calcium-enriched mixture. Iran Endod J.

[6] Prasad A, Pushpa S, Arunagiri D, Sawhny A, Misra A, Sujatha R (2015). A comparative evaluation of the effect of various additives on selected physical properties of white mineral trioxide aggregate. J Conserv Dent.

[7] Samiei M, Aghazadeh M, Lotfi M, Shakoei S, Aghazadeh Z, Vahid Pakdel SM (2013). Antimicrobial Efficacy of Mineral Trioxide Aggregate with and without Silver Nanoparticles. Iran Endod J.

[8] Eskandarinezhad M, Shahveghar-Asl N, Sharghi R, Shirazi S, Shakouie S, Milani AS (2017). Sealing efficacy of mineral trioxide aggregate with and without nanosilver for root end filling: An in-vitro bacterial leakage study. J Clin Exp Dent.

[9] Samiei M, Janani M, Asl-Aminabadi N, Ghasemi N, Divband B, Shirazi S (2017). Effect of the TiO2 nanoparticles on the selected physical properties of mineral trioxide aggregate. J Clin Exp Dent.

[10] Cinar C, Odabas M, Gurel MA, Baldag I (2013). The effects of incorporation of silver-zeolite on selected properties of mineral trioxide aggregate. Dent Mater J.

[11] Odabas ME, Cinar C, Akca G, Araz I, Ulusu T, Yucel H (2011). Short-term antimicrobial properties of mineral trioxide aggregate with incorporated silver-zeolite. Dent Traumatol.

[12] Cinar C, Ulusu T, Ozcelik B, Karamuftuoglu N, Yucel H (2009). Antibacterial effect of silver-zeolite containing root-canal filling material. J Biomed Mater Res B Appl Biomater.

[13] Thom DC, Davies JE, Santerre JP, Friedman S (2003). The hemolytic and cytotoxic properties of a zeolite-containing root filling material in vitro. Oral Surg Oral Med Oral Pathol Oral Radiol Endod.

[14] Basturk FB, Nekoofar MH, Gunday M, Dummer PM (2015). Effect of varying water-to-powder ratios and ultrasonic placement on the compressive strength of mineral trioxide aggregate. J Endod.

[15] Bernardi A, Bortoluzzi EA, Felippe WT, Felippe MC, Wan WS, Teixeira CS (2017). Effects of the addition of nanoparticulate calcium carbonate on setting time, dimensional change, compressive strength, solubility and pH of MTA. Int Endod J.

[16] Khatamian M, Irani M (2009). Preparation and characterization of nanosized ZSM-5 zeolite using kaolin and investigation of kaolin content, crystallization time and temperature changes on the size and crystallinity of products. Journal of the Iranian Chemical Society.

[17] Kachoei M, Nourian A, Divband B, Kachoei Z, Shirazi S (2016). Zinc-oxide nanocoating for improvement of the antibacterial and frictional behavior of nickel-titanium alloy. Nanomedicine (Lond).

[18] Samiei M, Aghazadeh M, Alizadeh E, Aslaminabadi N, Davaran S, Shirazi S (2016). Osteogenic/Odontogenic Bioengineering with co-Administration of Simvastatin and Hydroxyapatite on Poly Caprolactone Based Nanofibrous Scaffold. Adv Pharm Bull.

[19] Aminabadi NA, Behroozian A, Talatahari E, Samiei M, Sadigh-Eteghad S, Shirazi S (2016). Does prenatal restraint stress change the craniofacial growth pattern of rat offspring?. Eur J Oral Sci.

[20] Mirzakouchaki B, Shirazi S, Sharghi R, Shirazi S (2016). Assessment of Factors Affecting Adolescent Patients' Compliance with Hawley and Vacuum Formed Retainers. J Clin Diagn Res.

[21] Mirzakouchaki B, Shirazi S, Sharghi R, Shirazi S, Moghimi M, Shahrbaf S (2016). Shear bond strength and debonding characteristics of metal and ceramic brackets bonded with conventional acid-etch and self-etch primer systems: An in-vivo study. J Clin Exp Dent.

[22] Kachoei M, Mohammadi A, Esmaili Moghaddam M, Rikhtegaran S, Pourghaznein M, Shirazi S (2016). Comparison of multiple rebond shear strengths of debonded brackets after preparation with sandblasting and CO2 laser. J Dent Res Dent Clin Dent Prospects.

[23] Rodriguez-Lozano FJ, Garcia-Bernal D, Onate-Sanchez RE, Ortolani-Seltenerich PS, Forner L, Moraleda JM (2017). Evaluation of cytocompatibility of calcium silicate-based endodontic sealers and their effects on the biological responses of mesenchymal dental stem cells. Int Endod J.

[24] Saberi EA, Karkehabadi H, Mollashahi NF (2016). Cytotoxicity of Various Endodontic Materials on Stem Cells of Human Apical Papilla. Iran Endod J.

[25] Shi S, Bao ZF, Chen X, Zhang DD (2014). [Cytotoxicity of a novel endodontic treatment material iRoot BP Plus to human gingival fibroblasts]. Shanghai Kou Qiang Yi Xue.

[26] Bidar M, Eslami N, Naghavi N, Fasihi Z, Attaran Mashhadi N (2015). The effect of different concentrations of chlorhexidine gluconate on the compressive strength of mineral trioxide aggregate. J Dent Res Dent Clin Dent Prospects.

[27] Basturk FB, Nekoofar MH, Gunday M, Dummer PM (2013). The effect of various mixing and placement techniques on the compressive strength of mineral trioxide aggregate. J Endod.

